# Comparing Minimally Lethal Sampling Methods for Genomics in the Eastern Oyster (*Crassostrea virginica*)

**DOI:** 10.1002/ece3.73116

**Published:** 2026-02-24

**Authors:** Elisabeth Leung, Jessica Small, Katie E. Lotterhos

**Affiliations:** ^1^ Department of Marine and Environmental Sciences Northeastern University Marine Science Center Nahant Massachusetts USA; ^2^ Department of Evolution, Ecology, and Organismal Biology University of California Riverside California USA; ^3^ Virginia Institute of Marine Science William & Mary Gloucester Point Virginia USA

## Abstract

Understanding how selection acts on individual genotypes often requires genotyping at different life stages and tracking their survival under experimental conditions. However, in mollusks, such as the Eastern oyster ( *Crassostrea virginica* ), collecting sufficient high‐quality DNA without causing mortality remains challenging. While prior studies have explored different noninvasive genotyping methods, an optimal method hasn't been identified. Here, we tested different techniques by combining two minimally invasive accession techniques (relaxation, shell notching), four types of cell sampling (swab, mantle biopsy, hemolymph, and extrapallial fluid), and three preservation techniques (flash freezing, ethanol, and FTA card) using nine different treatment groups. We monitored mortality for 11 days after cell sampling and quantified the effects of each treatment on mortality rates, DNA quality, and DNA quantity. Our results show that mantle biopsies from relaxed oysters preserved in ethanol yielded DNA quantities comparable to the control groups and significantly higher than either of the liquid samples, while maintaining low post‐sampling mortality. Logistic model regression demonstrated that oysters from this treatment that are longer than 61 mm have a greater than 90% chance of survival. These findings establish a viable method for genotyping juvenile oysters while minimizing post‐sampling mortality, and will facilitate future studies on genomic selection, individual‐level survival responses to stress and disease, and repeated measures of gene expression.

## Graphical abstract

Non‐lethal genotyping of oysters is challenging due to the invasive nature of traditional tissue sampling methods. We compared four different sampling techniques on juvenile Eastern oysters, monitoring survival and DNA quality and quantity to identify the optimal approach. Mantle tissue sampling from relaxed oysters yielded the best balance of low mortality and high DNA yield, enabling future genomic selection studies and tracking individual stress responses without causing mortality.

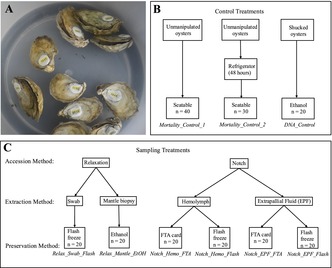

## 1 Introduction

Linking an individual's genetic makeup and fitness across their lifetime is essential to many ecological and evolutionary research avenues. These links support research on adaptation (Bordet et al. 2021; Harris et al. 2017), disease (Saura et al. 2019; Savage and Zamudio 2011), overall species health (Forsman 2014; Kristan et al. 2018; Meixner et al. 2015), and genomic selection (Goddard and Hayes 2007; Song et al. 2023; Zenger et al. 2019). A powerful way to correlate an individual's DNA sequence with survival is to sample tissue at the earliest possible stage in life. Unfortunately, tissue sampling often results in mortality for many invertebrate and small‐bodied organisms at the juvenile stage, thus limiting avenues of research. Thus, the ability to sample without causing mortality will enable broader studies of organismal fitness across taxa and environments, thus informing conservation and evolutionary science.

The ability of researchers to sample tissue without causing mortality varies among taxa and life stages. In plants and many vertebrate animals, genetic sampling can be performed early in life without causing mortality (e.g., from a leaf, hair, or fin clip) (De Kort et al. 2022). However, in animals with exoskeletons, such as mollusks, finding a non‐lethal sampling approach to enable sampling is challenging because accessing tissues usually requires opening the shell, causing irreparable damage to the muscle that closes their exoskeleton, effectively killing them. Another challenge for mollusks is finding a non‐lethal technique that is successful for all sizes, especially for juvenile life stages, which typically experience higher natural mortality rates (Gosselin and Qian 1997; Rossetto et al. 2012; Silina 1996).

Successful non‐lethal tissue sampling for mollusk genetics consists of three steps: accession, cell sampling, and preservation. Accession requires gaining access to the tissue inside the exoskeleton. This can be done lethally through shucking (forcefully opening the shell), or non‐lethally by relaxing (using liquid solutions to loosen the adductor tissue and allow access), or notching (creating a small slot in the shell to extract fluid containing genetic material). Notching is less invasive than shucking and involves creating a tiny slot on the edge of the shell to extract fluid (typically hemolymph) with a syringe (Chen et al. 2018; Donaghy et al. 2010; Mount et al. 2004). In Pacific oysters ( *Crassostrea gigas* ), a study that used notching to extract hemocytes did not report whether this process of fluid extraction affected oyster mortality (Donaghy et al. 2010). In contrast, tissue relaxation does not cause damage to the shell. Relaxation uses chemicals to cause the adductor muscle to slacken, allowing the valves to separate sufficiently for the inner soft body to be sampled. Prior studies have shown high efficacy of relaxants, like propylene phenoxetol, benzocaine, clove oil, magnesium chloride, and phenoxyethanol (Lellis et al. 2000; Mamangkey et al. 2009; Noble et al. 2009; Norton et al. 2000). However, research found negative side effects to the use of magnesium chloride (Azizan et al. 2021; Mamangkey et al. 2009). Epsom salt (magnesium sulfate) solutions have successfully relaxed oysters, although cells were not sampled from live oysters (Proestou et al. 2025). Notching is generally non‐lethal but limits sampling to fluids.

After the soft body is accessed, the next step is to sample cells. Common samples taken from mollusks include tissue biopsies, cell swabs, extrapallial fluid (EPF), and hemolymph. Tissue samples generally yield sufficient DNA for genotyping, but the effects of tissue removal on mollusk survival have not been well studied. Swabs from the adductor or viscera tissue have successfully yielded DNA in adult pearl oysters and pearl mussels (Karlsson et al. 2013; Massault et al. 2022). Liquid extraction offers an alternative to biopsies and swabs. EPF, a rapidly regenerated fluid between the shell and the mantle tissue, is promising for non‐lethal genetic sampling, but extraction from mollusks remains untested (Cameron 2020). Hemolymph is a fluid plasma that forms the basis for the circulatory system and can be sampled from inside the adductor muscle (Gustafson et al. 2005). Hemolymph has been extracted from mollusks with varied outcomes. Richling and Krause (2022) yielded wide ranges of 5–85 ng/μL, whereas Gustafson et al. (2005) and Caza et al. (2019) collected a consistent volume of hemolymph but did not report DNA yield or mortality. While no mortality was recorded in either field and lab experiments involving hemolymph extraction from adult mollusks, the effect of removing hemolymph from live juvenile oysters has not been well studied (Gustafson et al. 2005; Richling and Krause 2022).

Once tissues or cells are sampled, the next step is to preserve the cells for DNA extraction. Ideally, tissue samples collected for genetic work are rapidly frozen using liquid nitrogen and stored in a freezer until extraction (Dahn et al. 2022; Gutierrez‐Gongora et al. 2023) but may be impractical in field settings. Ethanol (95% pure) is generally recognized as a preservation method for long‐term storage and has high DNA yields (Karlsson et al. 2013; Massault et al. 2022), but it is not suitable for liquid biopsies because isolating the cells from the surrounding ethanol can be difficult. In addition, swab manufacturers usually do not recommend ethanol for swab preservation. For cells, another preservation method is with Flinders Technology Associates (FTA) cards, which have been shown to preserve small quantities of oyster hemocytes for up to 6 months and still yield DNA (Caza et al. 2019). FTA cards have been successful in other liquid DNA preservation, including for samples from blood, snail mucus, and algae (Burgos et al. 2019; Freshwater and Schmitt 2009; Leung 2024). In summary, there is an interdependence between the type of material collected and the preservation technique because some preservation techniques are better for specific types of material.

Although a range of non‐lethal methods have been trialed, key gaps remain. Many studies focus on either DNA yield or sampling success, but often don't quantify post‐sampling mortality or include juvenile individuals (Butt et al. 2008; Donaghy et al. 2010; Freshwater and Schmitt 2009; Gutierrez‐Gongora et al. 2023; Mamangkey et al. 2009; Massault et al. 2022). It remains unclear if these techniques would increase juvenile mortality, knowledge crucial for a wide range of applications, including genotyping of juvenile mollusks before selection experiments and linking individual genotypes to mortality for evolutionary studies.

This work aims to determine (1) the optimal method and (2) the minimum shell length for minimally lethal genotyping juvenile Eastern oysters ( *Crassostrea virginica* ). A successful minimally lethal method maximizes the quantity and quality of DNA extracted while minimizing individual mortality. Eastern oyster reefs offer invaluable ecosystem services, such as water filtration, coastal stabilization, and habitat for various organisms (Beck et al. 2011; Coen et al. 2007). These ecosystem services have been valued at up to $99,000 per hectare every year, while the oyster harvest of a healthy reef contributes another $50,000 annually (Grabowski et al. 2012). Despite their ecosystem importance and aquacultural relevance (Coen et al. 2007; Shumway et al. 2003), Eastern oyster reefs are declining (Beck et al. 2011). It is imperative to develop alternative, less harmful approaches for studying and conserving oysters to ensure the continued existence of oyster populations and the preservation of their ecosystems. A successful non‐lethal sampling method would enable future genetic research on oyster population health, including work on disease prevalence, climate adaptation, and demographic history.

## 2 Methods

**FIGURE 1:**
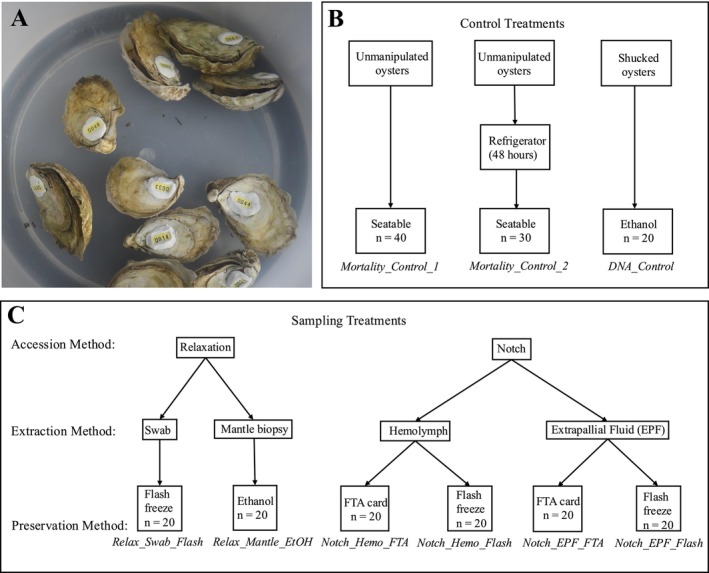
(A) Relaxed oysters in magnesium sulfate solution after 16 h with coral epoxy labels. (B) The three different control treatments for this experiment are shown with the treatment name (in italics) at the bottom of the figure. (C) The six treatment groups are broken down by accession, extraction, and preservation methods. Each treatment name (in italics near the bottom of the figure) is made up of the accession method, followed by the extraction method, and then the preservation method. “Relax” is short for the *Relaxation Protocol*, “Notch” is short for the *Notching Protocol*. “Swab” is short for *Swab Protocol*, “Mantle” is short for *Mantle Biopsy Protocol*, Hemo is short for *Hemolymph Protocol*, and EPF (abbreviated from Extrapalliel Fluid) is short for *Extrapalliel Fluid Protocol*. “Flash” is short for *Flash‐Freeze Protocol*, “EtOH” is short for *Ethanol Protocol*, and “FTA” is short for *FTA Cards Protocol*. A full description of the treatments is available in Table 1.

Juvenile oysters ranging from 30 to 60 mm were sourced from the Aquaculture Genetics and Breeding Technology Center (ABC) at the Virginia Institute of Marine Science (VIMS). Each oyster was cleaned using a 1% bleach solution, air‐dried for an hour, and labeled with a shellfish tag (Hallprint FPN 8 × 4 mm) using coral epoxy (Instant Ocean HoldFast Epoxy Stick) (Figure 1A). Shell width and length were measured for each oyster before the experiment (Kraeuter et al. 2007). To store the oysters before the experiment, the oysters were stored at 5°C for up to 48 h before experimentation. After each trial, the oysters were transferred to sea tables (flow‐through tanks filled with minimally filtered seawater from the adjacent York River), and mortality was monitored daily for 11 days. Daily mortality checks were conducted where each oyster was removed from the sea table briefly to check for shell gapes, which indicated loss of adductor muscle function and death.

### 2.1 Treatment Groups

We planned nine treatments to determine which protocol would have the best DNA yield with the lowest mortality (Figure 1). Each treatment had at least 20 individual oysters. Groups of 10 oysters per treatment were placed into separate trays in the sea tables. The treatments and short names are described in Table 1.

**TABLE 1:** Experimental treatment groups.

Treatment name	Response variable(s)	Description
*Mortality_Control_1*	Baseline mortality rate	30 individuals were placed in the sea tables at the beginning of the experiment
*Mortality_Control_2*	Baseline mortality rate after 48 h of refrigeration	30 individuals were placed in the sea tables after being refrigerated for 2 days
*DNA_Control*	Baseline DNA quantity & quality	Invasive tissue samples (mantle, adductor, and gill) from 30 individuals were stored in 95% molecular‐grade ethanol in 2 mL O‐ring vials and frozen at −20°C before transferring to a −80°C freezer
*Relax_Swab_Flash*	Mortality, DNA quantity & quality	20 individuals were treated using a relaxation protocol, followed by swabbing. Swabs were flash‐frozen and stored at −80°C
*Relax_Mantle_EtOH*	Mortality, DNA quantity & quality	20 individuals were treated using a relaxation protocol, followed by a mantle biopsy, with tissue preserved in 95% pure ethanol. Samples were frozen at −20°C before transferring to a −80°C freezer
*Notch_EPF_Flash*	Mortality, DNA quantity & quality	20 individuals notched, and extrapallial fluid (EPF) was extracted. EPF was flash‐frozen in liquid nitrogen and stored at −80°C
*Notch_EPF_FTA*	Mortality, DNA quantity & quality	20 individuals notched, and EPF was extracted. EPF was preserved on FTA cards stored at room temperature
*Notch_Hemo_Flash*	Mortality, DNA quantity & quality	20 individuals were notched, and hemolymph was extracted. Hemolymph was flash‐frozen in liquid nitrogen and stored at −80°C
*Notch_Hemo_FTA*	Mortality, DNA quantity & quality	20 individuals were notched, and hemolymph was extracted. Hemolymph was preserved on FTA cards stored at room temperature

#### 2.1.1 Control Groups

We established two control groups in our experiment: one to monitor mortality and the other to quantify the amount of DNA extracted.

*Mortality Control Groups* : We had two groups to control for mortality. *Mortality_Control_1*: At the beginning of the experiment, we labeled 30 individuals using coral epoxy and placed them into the sea tables. *Mortality_Control_2*: At the end of our trials, we added another 30 individuals to the sea tables that were stored in the refrigerator for 2 days. This was to monitor any effects of being in the refrigerator, controlling for the longest time of oyster refrigeration. *DNA_Control* : To establish a baseline for DNA quantification and quality, we sampled mantle, adductor, and gill tissues from 30 individuals and placed them into 2 mL O‐ring vials with 95% molecular‐grade ethanol. We stored the samples temporarily in a −20°C freezer before transferring them to a −80°C freezer.

### 2.2 Accessing the Oyster Tissue Within the Shell

Two methods were used to enter the oyster shell: creating a notch in the shell or relaxing the oyster. Creating a notch allowed for the extraction of fluid that contains genetic material (e.g., hemolymph or EPF), while relaxing the oyster allowed for tissue swabbing or direct sampling of the tissue. *Notch Protocol*: A variable‐speed dremel was used to make a small, V‐shaped notch in the oyster shell, just large enough for the needle (BD 23G PrecisionGlide Needle) to fit in at different angles. The size of the notch depended on how much new shell an oyster had recently produced, but was typically a few mm in size. The notch was created on the right valve of the oyster, approximately two‐thirds to the top of the shell. The needle, attached to a syringe (BD 1 mL Syringe), was used to extract fluid as described below for the *Hemolymph Protocol* and the *EPF Protocol*. *Relaxation Protocol*: For the relaxation protocol, 2 L of 1 μm filtered seawater was mixed with 100 g of Epsom salt until all the salt dissolved following the protocol by Proestou et al. (2025). A maximum of ten oysters were placed in each replicate 2 L container for 16 h before the cell sampling (see *Treatment Groups* for experimental design). Oysters were fully submerged in the Epsom salt solution and held at room temperature. During relaxation, containers were aerated using air stones and airlines to maintain oxygen levels.

### 2.3 Collection of Genetic Material

#### 2.3.1 Cell Collection for Notched Oysters

Cells were collected from notched oysters by sampling hemolymph or EPF fluids. For the preservation of fluids, we split the samples and placed half in liquid nitrogen and the other half in FTA cards (see below). *Hemolymph Protocol*: We inserted the needle through the notch diagonally down into the adductor muscle, which we identified through increased resistance. We collected 125 to 250 μL of hemolymph. *EPF Protocol*: We inserted the needle into the notch until it reached the mantle cavity of the shell and collected 80 to 500 μL of EPF from each oyster. If necessary, we removed and reinserted the needle at multiple angles for small oysters to obtain a minimum fluid amount of 80 μL.

#### 2.3.2 Cell Collection for Relaxed Oysters

Cells were collected from relaxed oysters by taking a mantle biopsy or swabbing the mantle tissue. *Mantle Biopsy Protocol*: We used forceps and scissors to remove a small piece (~10–20 mg) of the mantle wrapped around the adductor muscle. We sampled the distal edge of the mantle tissue, approximately 2–3 mm away from the visible edge of the adductor muscle. After each sample was acquired, we sterilized the forceps and scissors using an ethanol lamp. *Swab Protocol*: Using an OmniSwap (Qiagen; WB100035), we swabbed in a circular motion, starting at the top layer of the mantle and moving down to the mantle around the adductor, then to the mantle's bottom layer, and then reversing this path. The motion was repeated five times for each sampled individual.

### 2.4 Preservation of Genetic Material

We used three preservation protocols for the genetic samples: ethanol, flash‐freezing, and FTA cards. *Ethanol Protocol*: The mantle sampled from the relaxed oysters was preserved with 95% molecular‐grade ethanol. It was temporarily stored in a −20°C freezer and then transferred to a −80°C freezer. *Flash‐Freeze Protocol*: Liquid nitrogen was used to flash‐freeze half of each EPF or hemolymph sample from the notched oysters. After the samples were flash‐frozen, they were stored in a −80°C freezer. Additionally, the swabs used on the relaxed oysters were flash‐frozen and then stored in a −80°C freezer. *FTA Cards Protocol*: The remaining half of the EPF and hemolymph samples were stored on QIAcard FTA Classic Cards (Qiagen; WB120305). Following the manufacturer's instructions, we put up to 125 μL of fluid on each circle in the FTA card. FTA cards were stored in WhirlPak bags with color‐indicating silica beads (desiccants that absorb moisture and change color to indicate saturation) following the manufacturer's recommendations.

### 2.5 DNA Extraction

A Qiagen DNeasy Blood & Tissue kit (Qiagen; 69504) was used for all DNA extractions, but with slight modifications for different cell types or preservation methods. The manufacturer's instructions were used for the control and the mantle biopsy groups with 20–30 mg of tissue, as well as the swabs (swabs were horizontally cut in half before extraction so that they could be submerged fully in the buffer). We modified the protocol for the EPF and Hemolymph samples by using 200 μL of the flash‐frozen sample as input (based on preliminary trials of 20, 100, and 200 μL that showed the latter amount yielded the most DNA) but otherwise followed the manufacturer's instructions. For the Whatman FTA Cards, we used a 6.0 mm biopsy punch (Cenmed PMD Sterile, Disposable Biopsy Punches) to cut a disk from the middle of the card. Per recommendation from Sigma Aldrich, 280 μL of the ATL buffer was added to the disk for extraction, and the remainder of the manufacturer's instructions were followed (Merck n.d.).

### 2.6 DNA Quantification and Quality Assessment

We used a Qubit 3.0 Fluorometer to quantify DNA yield from the extractions. We used the High Sensitivity Kit (ThermoFisher; Q32851) for samples with a yield between 0 and 20 ng/μL and the Broad Range Kit (ThermoFisher; Q32850) for all samples with a yield higher than 20 ng/μL.

To test the quality of the DNA, we ran 3 μL of each DNA extract on a 1% agarose gel (0.6 g of agarose with 60 mL of 1× TAE buffer), stained with GelRed at 130 V for 30 min. Gels were visualized, and images were captured on a BioRad Gel Doc XR+ Molecular Imager machine. We categorized each sample on the gel into one of four levels: a bright band greater than 3000 bp with a slight smear (level 1), a completely smeared sample (level 2), a barely visible gel (level 3), or no band (level 4).

### 2.7 Statistical Analyses

#### 2.7.1 Oyster Size Distribution Across Treatments

Although we randomly assigned oysters to treatments, we conducted a one‐way ANOVA of oyster length and treatment to assess potential sampling bias. We used the “aov” function in R to perform an analysis of variance with the treatment groups as the explanatory variable and the length of the oysters as the response variable, and a Tukey HSD test to examine pairwise differences among treatments (“TukeyHSD” in R).

#### 2.7.2 Comparison of Survival Between Control Groups

First, we tested the null hypothesis that the probability of survival was equal between the two control groups (*Mortality_Control_1* and *Mortality_Control_2*) with a binomial test (function “binom.test()” in R with the number of successes set to the number of surviving oysters after 11 days and the sample size the total number of oysters in each control group). The alternate hypothesis was that the survival of *Mortality_Control_1* and *Mortality_Control_2* was not equal.

#### 2.7.3 Survival Through Time

To ensure the length of the experiment was sufficient, we conducted a survival analysis. Survival data were converted into a numerical binary variable, with 1 representing alive and 0 representing dead after 11 days. Using all samples, we created a survival object with the binary survival data and the lifespan of each oyster, with no grouping term, using the “survfit” function from the “survival” package in R. The survival object was plotted in a Kaplan–Meier survival curve.

#### 2.7.4 Effect of Treatment and Size on Survival

To test the effects of treatment and oyster size on mortality, we created a logistic survival model with survival (binary) as the response variable, treatment as an explanatory factor, and length as an explanatory covariate. We implemented the model using the “glm” function in R and specified family = binomial (link = “logit”).

#### 2.7.5 DNA Quantity Analysis

To test if there was a statistical difference in the total amount of DNA among treatment groups (DNA_Control, Relax_Swab_Flash, Relax_Mantle_EtOH, Notch_Hemo_Flash, Notch_EPF_Flash), we conducted an ANOVA test using the “aov” function in R. The explanatory variable was the different treatment groups, and the response variable was the DNA concentration. The null hypothesis was that the treatment groups had no difference in DNA concentration. After a significant ANOVA result, we conducted post hoc comparisons using Tukey's HSD test to identify which treatment groups differed significantly in DNA concentration while controlling for multiple comparisons.

#### 2.7.6 DNA Quality Analysis

To address the question of which method had the highest DNA quality, we scored the gels on a range from 1 to 4, with 1 being the highest quality. All scoring was done by the same person for consistency and to minimize variability. For statistical analysis, we performed a chi‐square test to test the null hypothesis of no association (i.e., independence) between the gel categorization scores and treatment groups using the “chisq.test()” in R. The alternative hypothesis was that the different treatment groups and the gel categorization scores were not independent.

## 3 Results

Across all the relaxation containers, 100% of the oysters were gaping by approximately 5 mm after 16 h in the magnesium sulfate solution, which was sufficient to biopsy or swab. Although oysters were randomly distributed to treatments, an ANOVA showed significant differences in shell length among treatments (ANOVA, *F* = 2.938, *p*‐value = 0.00966). Although posthoc comparisons using TukeyHSD failed to reject the null hypothesis of equal lengths among pairwise comparisons (*p*‐value > 0.05; likely because there is less power than with an ANOVA), the *Relax_Mantle_EtOH* treatment had included slightly smaller oysters relative to the other treatments (the mean lengths of oysters in the *Relax_Mantle_EtOH* treatment were 39.11 mm, while the oysters in the treatment *Notch_EPF_Flash* had the highest average length at 46.05 mm). In these juvenile oysters, small differences in oyster size of ~5 mm are biologically relevant because size significantly determines oyster survival after biopsy treatment (see Section 3.2). A one‐way ANOVA on treatment type and oyster width failed to reject the null hypothesis of equal widths (ANOVA, *F* = 0.673, *p*‐value = 0.671), so oyster widths did not differ across treatment groups. We included width as a secondary measurement here to further confirm the random size distribution.

### 3.1 Exclusion of Treatments

After testing DNA extraction from FTA cards (10 samples each for *Notch_EPF_FTA* and *Notch_Hemo_FTA*), we obtained a very low yield of DNA (less than 1 ng/μL per sample) compared to the other methods. As a result, both methods were excluded from further analyses due to insufficient DNA yields.

### 3.2 Controls and Treatment Survival

**FIGURE 2:**
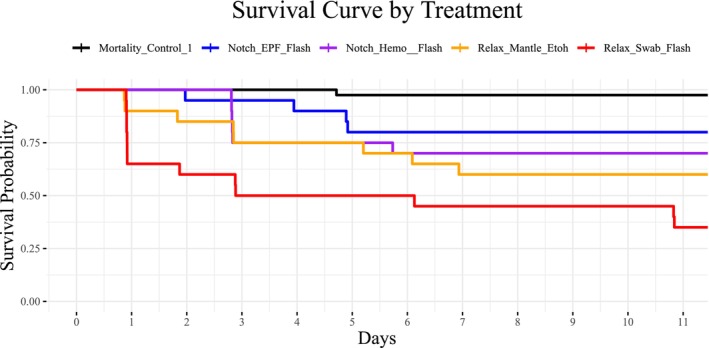
A Kaplan–Meier plot showing the probability of survival throughout the experiment. All sampling treatment groups (except the excluded FTA groups) and *Mortality_Control_1* are included on this plot.

Across all treatments and *Mortality_Control_1*, oyster survival declined most sharply in the first 3 days after treatment, but stabilized by Day 8 with only one additional mortality over the following 3 days (Figure 2). Cumulative survival from the unmanipulated control oysters was high (> 95%). To assess the effects of refrigeration, we compared the proportion of unmanipulated oysters that survived after 11 days from *Mortality_Control_1* (no refrigeration) and *Mortality_Control_2* (prior refrigeration for 24 h). We concluded that the proportion of survival from *Mortality_Control_1* and *Mortality_Control_2* was statistically equivalent (binomial test, *p*‐value = 1), and so we combined them for most subsequent analyses (hereafter: *Mortality_Control*). However, analyses involving oyster length are based on only the *Mortality_Control_1* individuals because we did not measure the length of oysters in *Mortality_Control_2*.

**FIGURE 3:**
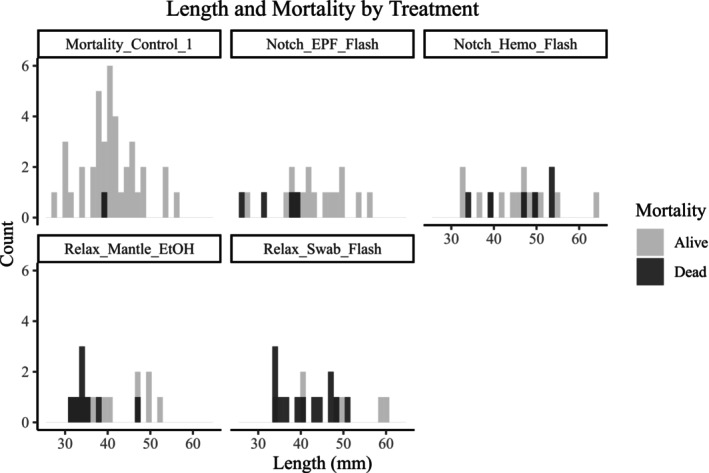
A ridge plot shows the length distribution for alive and dead oysters for each treatment. Each treatment name on the *Y*‐axis is made up of the accession method, followed by the extraction method, and then the preservation method. “Relax” is short for the *Relaxation Protocol*, and “Notch” is short for the *Notching Protocol*. “Swab” is short for *Swab Protocol*, “Mantle” is short for *Mantle Biopsy Protocol*, Hemo is short for *Hemolymph Protocol*, and EPF (abbreviated from Extrapalliel Fluid) is short for *Extrapalliel Fluid Protocol*. “Flash” is short for *Flash‐Freeze Protocol*, “EtOH” is short for *Ethanol Protocol*, and “FTA” is short for *FTA Cards Protocol*. A full description of the treatments is available in Table 1.

**FIGURE 4:**
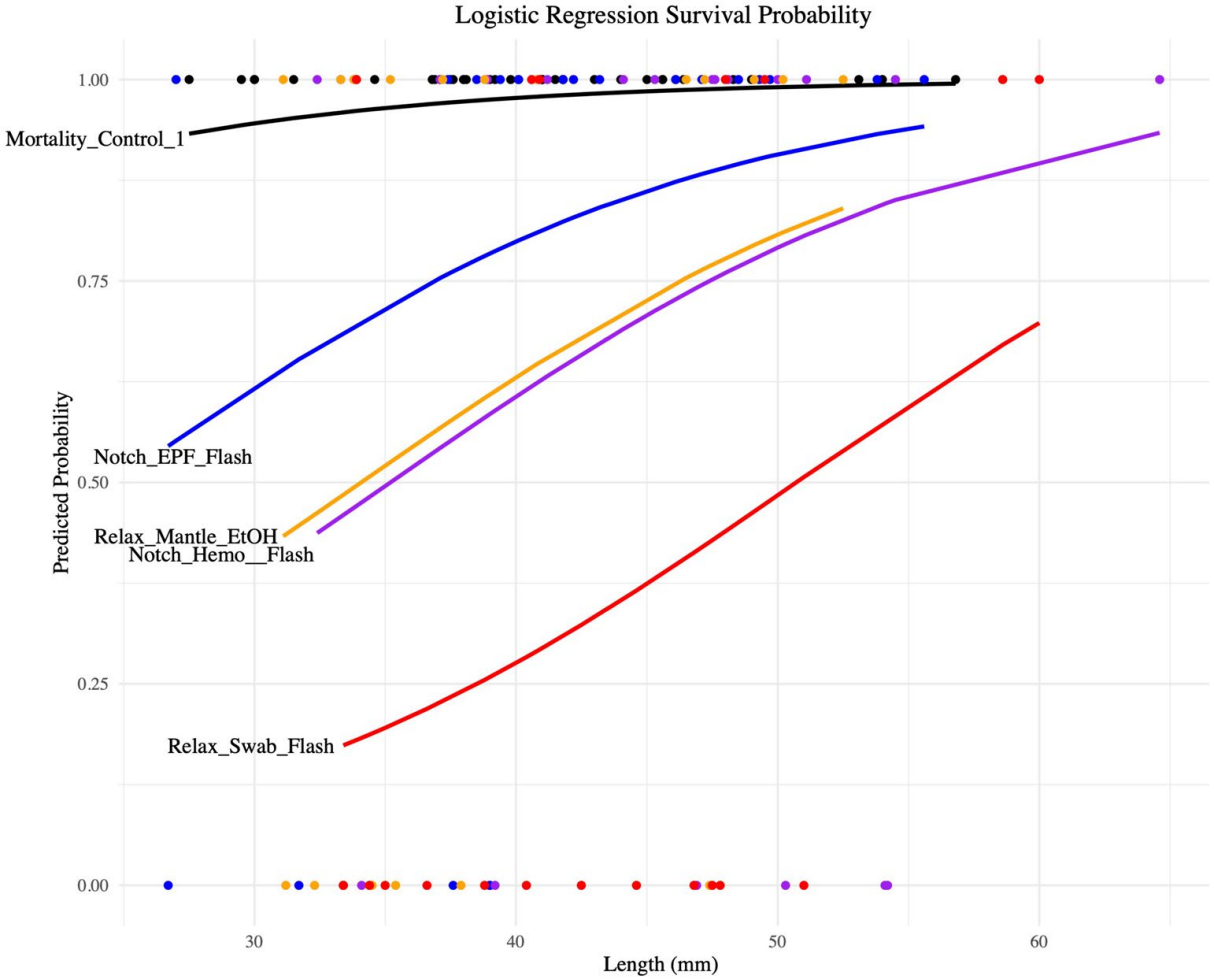
A logistic regression plot shows each treatment's predicted probability based on length. Each treatment name on the plot is made up of the accession method, followed by the extraction method, and then the preservation method. “Relax” is short for the *Relaxation Protocol*, and “Notch” is short for the *Notching Protocol*. “Swab” is short for *Swab Protocol*, “Mantle” is short for *Mantle Biopsy Protocol*, Hemo is short for *Hemolymph Protocol*, and EPF (abbreviated from Extrapalliel Fluid) is short for *Extrapalliel Fluid Protocol*. “Flash” is short for *Flash‐Freeze Protocol*, “EtOH” is short for *Ethanol Protocol*, and “FTA” is short for *FTA Cards Protocol*. A full description of the treatments is available in Table 1. The FTA treatments (*Notch_EPF_FTA*, *Notch_Hemo_FTA*) were excluded due to low DNA yield and therefore not being a viable option.

Logistic regression revealed that survival probability was significantly influenced by both treatment and oyster length (Table 2). Across all treatments, larger oysters consistently exhibited higher survival probabilities (Figures 3 and 4). Treatment effects were significant, with manipulated oysters having a significantly lower probability of survival than unmanipulated controls (Table 2, Figures 3 and 4). The highest cumulative survival was exhibited by *Mortality_Control_1* (97.14%), and *Relax_Swab_Flash* exhibited the lowest (35%) (Figures 3 and 4).

**TABLE 2:** The *p*‐values for each group from the logistic regression. Results show that the length and all the treatments are statistically significant (< 0.05).

Group	Null hypothesis	*p*
Intercept	The log odds of survival are zero when the length is zero	0.92707
Length	There is no relationship between length and the log odds of survival (controlling for treatment)	0.01051
*Notch_EPF_Flash*	No difference in the log odds of survival compared to the *Mortality_Control* group	0.04254
*Notch_Hemo_Flash*	No difference in the log odds of survival compared to the *Mortality_Control* group	0.00415
*Relax_Mantle_EtOH*	No difference in the log odds of survival compared to the *Mortality_Control* group	0.00408
*Relax_Swab_Flash*	No difference in the log odds of survival compared to the *Mortality_Control* group	4.5 × 10^−5^

Using the “predict” function, we determined the lengths of oysters for each treatment that would result in a 10% mortality rate: 60 mm for the *Notch_Hemo_Flash* treatment, 50 mm for the *Notch_EPF_Flash* treatment, 59 mm for the *Relax_Mantle_EtOH* treatment, and 76 mm for the *Relax_Swab_Flash* treatment.

### 3.3 DNA Quantity and Quality

**FIGURE 5:**
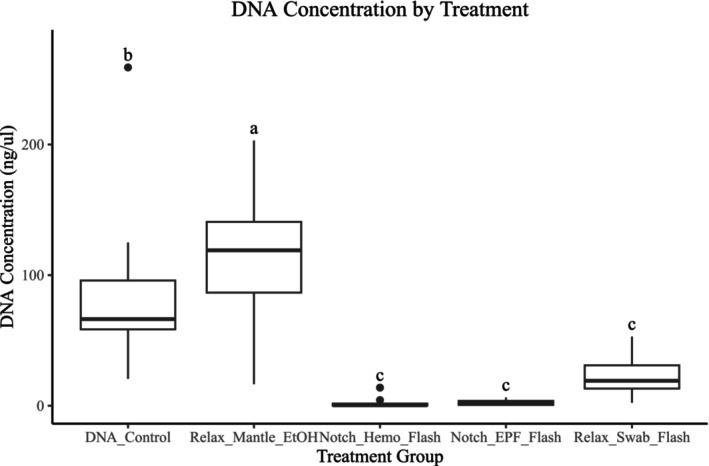
A boxplot showing the DNA yield in nanograms per microliter for each treatment group. The FTA treatments (*Notch_EPF_FTA*, *Notch_Hemo_FTA*) averaged less than 1 ng/μL, so they were excluded. Letters indicate a significant difference between treatments (*p* < 0.05) based on TukeyHSD post hoc tests.

We assessed DNA extracted from the different treatment groups for DNA quality and quantity. An ANOVA on the DNA per microliter per sample as a function of treatment revealed significant differences across treatment groups (Figure 5, ANOVA, *F* = 56.43, *p*‐value = < 2 × 10^−16^). *Notch_EPF_Flash* and *Notch_Hemo_Flash* had significantly lower total DNA yield than the *DNA_Contro*l control group (Figure 5, Tukey HSD, *p*‐value < 0.000001). On the other hand, *Relax_Mantle_EtOH* had significantly higher total DNA yield compared to *DNA_Control*, *Notch_EPF_Flash*, and *Notch_Hemo_Flash* (Figure 5, Tukey HSD, *p*‐value = 0.0017837, *p*‐value < 0.000001, *p*‐value < 0.000001, respectively). Additionally, *Relax_Swab_Flash* showed significantly lower total DNA yield than the control group and *Relax_Mantle_EtOH* (Figure 5, TukeyHSD, *p*‐value < 0.000001).

**FIGURE 6:**
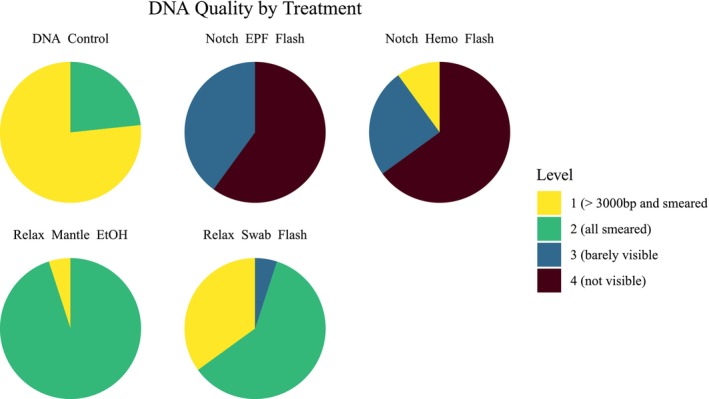
Pie charts for each treatment showing DNA quality scores. Based on the gel results, there are four score levels: Level 1 indicates bands greater than 3000 bp with a slight smear, Level 2 signifies a completely smeared sample, Level 3 corresponds to a barely visible gel, and Level 4 denotes no visibility. Level 1 represents the highest quality, while Level 4 indicates the lowest quality. The FTA treatments (*Notch_EPF_FTA*, *Notch_Hemo_FTA*) did not yield more than 60 ng, so they are not shown.

A chi‐squared test between treatment groups and gel categorization scores found a highly significant association between these two categorical variables (chi‐square test, *p*‐value = 6.53 × 10^−24^, Figure 6). The *DNA_Control* group demonstrated the highest quality DNA, with most samples having bands greater than 3000 bp, with a minority of smeared samples, while *Relax_Mantle_EtOH* had predominantly smeared samples with few samples greater than 3000 bp. High molecular weight bands signify that the DNA quality is not degraded and is suitable for downstream analyses, like sequencing. Smearing occurs when the bands indicating DNA length are not distinct, suggesting fragmentation and degradation. In contrast, the swab samples (*Relax_Swab_Flash*) had substantial smearing, and the hemolymph and EPF samples (*Notch_Hemo_Flash* and *Notch_EPF_Flash*) had bands that were barely or not visible (Figure 6).

## 4 Discussion

**FIGURE 7:**
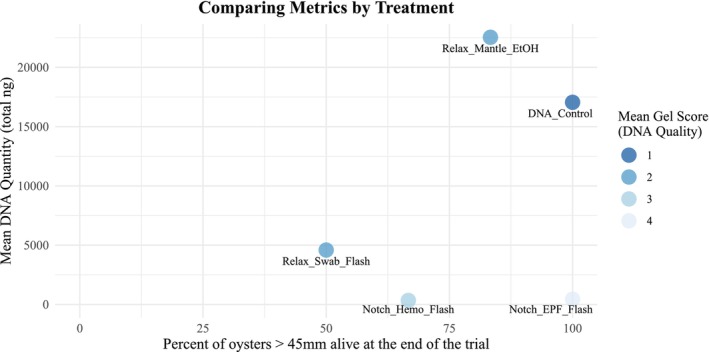
A scatterplot combining the different metrics for each treatment group that we had DNA metrics for. The *X* axis is the percent alive for each treatment group of oysters greater than 45 mm at the end of the trial (Day 11 of mortality checks). The *Y* axis shows the mean total DNA quantity, and the color of the points is the mean gel score with 1 representing the highest quality, while 4 indicates the lowest quality.

This study, focusing on the Eastern oyster, investigated the effects of different minimally‐lethal DNA sampling techniques on subsequent mortality, DNA quality, and quantity. Although sampling EPF resulted in the lowest post‐sampling mortality of the manipulations, the yield and quality of extracted DNA were low. Relaxed oysters that were sampled with swabs had the highest mortality, although DNA yield was slightly higher and of better quality than the EPF and hemolymph samples. Mantle‐sampled relaxed oysters experienced relatively low mortality (similar to notching and hemolymph extraction) and were the only samples that had DNA quantity comparable to the controls, also with sufficient quality (Figure 7). The DNA quality of the mantle biopsies was slightly lower than that of the controls, possibly due to degradation or bacteria during the relaxation protocol. Thus, the most promising procedure is the mantle biopsy from relaxed oysters because it balances a high yield of quality DNA with minimal mortality.

We found that small differences in size can translate to large differences in survival, which aligns with prior work that smaller juvenile mollusks are more vulnerable to stress (Gosselin and Qian 1997; Rossetto et al. 2012; Silina 1996). Survival after sampling increased with size, and at very small sizes (< 50 mm), the cell accession and tissue sampling drastically increased the probability of mortality. When taking mantle tissue from the relaxed oysters, oysters longer than 59 mm (about 1 year old) were predicted to have less than 10% mortality. This pattern is consistent in other studies linking size and survival in mollusks. In out‐planting experiments, longer soft‐shell clams and Manilla clams had higher survival than smaller individuals (Beal and Kraus 2002; Cigarría and Fernández 2000). Similarly, larger juvenile oysters had increased overwintering survival (Hidu et al. 1988). A recent study using geoducks found that a condition index based on shell length and wet weight was a better predictor of survival than only using shell length (Rimmer et al. 2025). However, shell length was still positively correlated with survival, but the addition of wet weight is particularly important for burrowing mollusks that need soft‐tissue reserves for burrowing and tolerating hypoxia (Rimmer et al. 2025).

With the techniques from this study, juveniles can be genotyped for SNP‐based breeding values and parentage while remaining alive for subsequent growth, survival, or disease‐challenge evaluations, removing the need for lethal genotyping. The techniques from this study progress genomic selection frameworks already established in other agriculture and livestock species (Bernardeli et al. 2025; Esrafili Taze Kand Mohammaddiyeh et al. 2023; Mastrodomenico et al. 2019; Ning et al. 2022; Parveen et al. 2023) to be applied directly to oysters and other aquaculture species. Genomic selection in aquaculture has been predominantly focused on fish, including the Japanese flounder (Lu et al. 2020), Atlantic salmon (Tsai et al. 2016), and rainbow trout (Kudinov et al. 2024) using fin clips for genotyping. Our method will enable similar approaches in mollusks.

Our minimally lethal sampling method enables repeated sampling of the same individual to track responses to environmental stressors over time. In other species, like humans, it has been found that gene expression changes over a lifespan (Harris et al. 2017). In mollusks, most studies have relied on lethal sampling at specific time points, preventing tracking the same individual over time (De Wit et al. 2018; Downey‐Wall et al. 2020; McFarland et al. 2022). With the mantle biopsy method, researchers could track gene expression responses in mantle tissue within an individual before and after a stress event (e.g., disease exposure), leading to a more precise study of responses while controlling for individual variability. We did not evaluate the survival of oysters after multiple relaxation and biopsy treatments, and so this is an important avenue for future research.

These techniques will advance the study of adaptation, especially understanding differential mortality based on an individual's genotype. Most studies have focused on populations before and after a selection event (Barrett et al. 2019; Gompert et al. 2014; Pespeni et al. 2013), but likely have low statistical power to detect changes in allele frequency (Albecker et al. 2021). However, non‐lethal sampling and genotyping at a juvenile stage enable the direct association of individual genotypes to individual fitness outcomes, leading to increased statistical power for discovering the genetic basis of adaptation compared to studies based on pools of individuals.

The minimally lethal sampling method developed in this study provides a foundation for cell sampling from different tissue types. Although our study focused on mantle tissue, future research should investigate the efficacy of sampling other tissues, such as the gill, adductor, and gonad. Gill tissue is commonly used for disease testing in aquaculture (Birlanga et al. 2022; Cho et al. 2025; Pires et al. 2022; Vallarino et al. 2024) and diseases can cause significant mortality in populations and decrease aquaculture production. Non‐lethal gonad sampling could provide valuable information on reproductive status, fertility, and the detection of reproductive disorders, enabling better management of population health in aquaculture (Blazer 2002; Boles et al. 2023).

Some limitations should be considered when applying these methods. Our experiment occurred in an aquaculture facility under controlled conditions, and mortality rates of oysters placed directly into the wild or a stressful treatment may differ. We recommend monitoring individuals for at least 5–7 days post‐biopsy, during which the majority of mortality occurred in our study. In addition, mortality rates may be affected by the prior exposure of oysters to stress or disease, or unknown carryover effects. Carryover effects in oysters have been documented in multiple oyster species. For example, ocean acidification exposure produces larger larvae and faster development in the Sydney rock oyster, but causes reduced larval shell growth and slower development in the Olympia oyster (Hettinger et al. 2012; Parker et al. 2015). Similarly, Eastern oysters showed negative carryover effects that decreased the ratio of tissue to shell growth (Donelan et al. 2021). Disease history is another critical factor as diseases have caused significant mortality across oyster populations historically. Oyster populations experienced mortality rates of 50%–60% in the first 2 years following MSX introduction (Andrews 1966). A long‐term study in the Delaware River showed that Dermo causes annual mortality rates exceeding 20% (Powell et al. 2008). Understanding these potential influences on mortality is crucial for ensuring long‐term survival of individuals after minimally invasive sampling. Overall, our study proposes a novel minimally non‐lethal genotyping method, providing an opportunity for individual‐level insights into links between the genotype and fitness, environmental adaptations, and responses to selection. The application of this technique will facilitate advances in evolutionary biology, but also in aquaculture by tracking desirable traits (such as disease resistance) and in conservation by reducing the amount of lethal sampling.

## Author Contributions

**Elisabeth Leung:** data curation (lead), methodology (equal), writing – original draft (lead), writing – review and editing (equal). **Jessica Small:** funding acquisition (supporting), methodology (equal), resources (equal), writing – review and editing (equal). **Katie E. Lotterhos:** funding acquisition (lead), methodology (equal), project administration (lead), resources (equal), writing – review and editing (equal).

## Conflicts of Interest

The authors declare no conflicts of interest.

## Data Availability

The data from this project is available on the GitHub repository for peer review at https://github.com/elisabethleung/non_lethal_oysters. Data are archived in BCO‐DMO under the project https://www.bco‐dmo.org/project/876610, and the reproducible code in the GitHub repo is archived with Zenodo and is available at www.doi.org/10.5281/zenodo.18654417.
